# Catalpol Protects Against High Glucose-Induced Bone Loss by Regulating Osteoblast Function

**DOI:** 10.3389/fphar.2021.626621

**Published:** 2021-03-10

**Authors:** Lu Zhao, Wei Du, Dandan Zhao, Xueyan Ji, Yanfei Huang, Yong Pang, Kaijin Guo, Xiaoxing Yin

**Affiliations:** ^1^ Jiangsu Key Laboratory of New Drug Research and Clinical Pharmacy, Xuzhou Medical University, Xuzhou, China; ^2^ Department of Emergency Medicine Center, The Affiliated Hospital of Xuzhou Medical University, Xuzhou, China; ^3^ Department of Orthopedics, The Affiliated Hospital of Xuzhou Medical University, Xuzhou, China

**Keywords:** diabetic osteoporosis (DOP), catalpol, osteoblast, differentiation, migration

## Abstract

**Objective:** The overall objective of this study was to investigate the effects of catalpol on bone remodeling of diabetic osteoporosis by regulating osteoblast differentiation and migration.

**Method:** Using a murine model of diabetic osteoporosis, to detect the protective effects of catalpol on bone loss, architectural deterioration of trabecular bone and bone metabolism biomarkers were tested. A model of MC3T3-E1 cells was established by treatment with high glucose; the regulatory role of catalpol in the differentiation and migration was tested by Western blot, ALP staining, and Alizarin Red staining.

**Results:** Catalpol treatment markedly ameliorated trabecular bone deterioration by reducing degenerative changes of the trabecular structure by improving the bone formation marker levels of ALP, osteopontin, type I collagen, and osteocalcin, as well as the level of OPG/RANKL. Catalpol enhanced cell motility and scattering following gap formation of MC3T3-E1 cells.

**Conclusion:** The results indicated that catalpol exhibits a protective effect against diabetic osteoporosis by regulating the differentiation and migration of osteoblast.

## Introduction

Accumulating evidence has shown that diabetes is associated with an increased risk of osteoporosis and fragility fractures. Therefore, the presence of low bone mineral density (BMD) and delay in fracture healing in diabetic patients have begun to receive more attention (Kanazawa et al., 2018). Research proves that patients with type 2 diabetes have greater trabecular but lower cortical BMD (Kanazawa et al., 2018). In the progression of diabetes, hyperinsulinemia as well as the relative impairment of glucose metabolism suppresses bone formation, which explains the discrepant results of BMD (Vestergaard, 2007). The adverse impact of hyperglycemia and oxidative stress induced by high glucose on bone formation plays a critical part in the etiology of the bone loss of diabetic osteoporosis (Pereira et al., 2017). Under conditions of high glucose, hyperglycemia leads to excessive reactive oxygen species (ROS), disrupts the cellular oxidant–antioxidant balance, and causes oxidative damages of osteoblasts (King et al., 2016). In addition, high-level blood glucose and advanced glycation end-products (AGEs), activation of protein kinase C isoforms, glucose autoxidation, and mitochondrial overproduction of superoxide contribute to increased ROS generation in osteoblast (Dong et al., 2017).

It has been proved by various researches that antidiabetic drugs cannot effectively reduce bone loss, and even result in degeneration of bone quality. The use of thiazolidinediones and insulin improves the risk of fracture, while metformin and sulfonylureas may have a neutral or negative effect on bone in diabetic persons (Palermo, D’Onofrio et al., 2015; Chandran, 2017). Therefore, a natural compound with bone-protective properties may have the advantage to treat bone loss induced by high glucose.

A vast amount of clinical and experimental researches has demonstrated that *Rehmannia glutinosa* is an important traditional Chinese medicine to treat bone loss by improving BMD in osteoporosis patients (Liu et al., 2017). In addition, *Rehmannia glutinosa* has been widely used in herbal formulas to treat diabetes and complicating disease (Ma et al., 2016). Catalpol, a kind of iridoid glycosides, is the main active chemical constituent of the root of *Rehmannia glutinosa* Libosch and is now widely used for the treatment of nervous system diseases, cardiovascular disease, and tumor diseases, as well as diabetes and complicating disease (Lai et al., 2015; Jiang et al., 2018; Yan et al., 2018). Extract of *Rehmannia glutinosa* can improve total femur BMD and trabecular structure of diabetic rats induced by streptozotocin mainly by promotion bone formation effects. The present study is designed to study the effects of catalpol on the bone loss of diabetic osteoporosis mice and osteogenic differentiation and migration of MC3T3-E1 cells.

## Materials and Methods

Catalpol was purchased from Nanjing Jingzhu Biotechnology Co., Ltd. (purity > 99%). 55% high-fat diet was purchased from Shanghai SLAC Company. The OPN (ab214050), type I collagen (ab34710), osteocalcin (ab93876), OPG (ab203061), and RANKL (ab45039) antibody were purchased from Abcam (Cambridge, MA). Glucose (47829), streptozotocin (v900890), and all other chemicals were purchased from Sigma.

### Animal Feeding Study

Mice (clean level) were bought from Shanghai Slyke Experimental Animal Co., Ltd., China, and housed in specific pathogen-free conditions with ad libitum access to food and water at the Jiangsu Key Laboratory of New Drug Research and Clinical Pharmacy. Forty 7-week-old male ICR mice were divided into four groups randomly (*n* = 10 per group): control group; model group (diabetic osteoporosis group); low-dose group (diabetic osteoporosis group with 30 mg/kg catalpol); and high dose group (diabetic osteoporosis group with 90 mg/kg catalpol). The type 2 diabetes model was induced through a high-fat diet and intraperitoneal injection of low-dose streptozotocin. The mice (except the normal group) were fed with 55% high-fat diet fed (4 weeks) and then intraperitoneally injected with streptozotocin (0.5% of STZ solution was prepared in 0.1 mol citrate buffer, using 1 ml syringe) at a dose of 40 mg/kg for five consecutive days to build type 2 diabetes osteoporosis model (blood glucose level of >16.0 mmol/L; FBG more than 13 mmol/L) (Xie et al., 2018). The health status of all the mice was observed during the modeling. The success rate of modeling was 100%, and the injured mice did not exhibit death following the impact of STZ. After 12 weeks of intragastric administration of vehicle control or catalpol, serum and femurs were immediately harvested for a variety of analyses.

### Cell Culture and Treatment

MC3T3-E1 cells were purchased from the typical Culture Collections Committee cell library of the Chinese Academy of Sciences (Shanghai, China). After resuscitation, MC3T3-E1 cells were maintained in minimum essential medium-α (MEM-α) (HyClone, Logan, UT, United States) containing 10% fetal bovine serum (FBS) in a humidified atmosphere of 5% CO_2_ in air at 37°C. The mineralizing medium was used to induce osteogenic differentiation containing 10% fetal bovine serum, 50 mg/ml ascorbic acid, 10 mM *ß*-glycerophosphate phosphate, and 10^−8^ M dexamethasone (Dong et al., 2017). In a high glucose experiment, 25.5 mM glucose was added to *a*-MEM to mimic high glucose concentration while 25.5 mM mannitol was used as an osmotic control for high glucose conditions (Zhou et al., 2019). MC3T3-E1 cells (reached approximately 80%) were maintained in mineralizing medium and divided into the following four groups: normal control group, high-glucose group, and catalpol groups (1 and 10 μM). To test the osteogenesis, Western blot analysis of the osteoblastic markers, ALP activity assay, Alizarin Red staining, and other assays were performed (Ho-Shui-Ling et al., 2018).

### Alkaline Phosphatase Activity Assay and Staining

MC3T3-E1cells were suspended in *a*-MEM containing 10% FBS and plated in 12-well culture plates at a density of 5 × 10^4^ cells per well. When the cells reached approximately 80% of the well, the cells were maintained in mineralizing medium and cultured with catalpol (1 and 10 μM). After 2 h, the cells were treated with high glucose (25.5 mM glucose in the culture medium) to mimic the diabetic state for 7 days (Dong et al., 2017). The medium was changed every 3 days. ALP activity was determined in the cell culture supernatants according to the manufacturer’s instructions of ALP activity assay kit (Jiancheng, Nanjing, China). The test samples and buffers were prewarmed to room temperature. 50 µl/well of *p*-nitrophenol was added to a 96-well plate, and 100 µl of samples was mixed well with the substrate, then incubated at 37°C for 10 min, and added 100 µl stop solution. The color change of *p*-NPP to *p*-nitrophenol was measured at 405 nm using a microplate reader (Li et al., 2012).

### Alizarin Red Staining

MC3T3-E1 cells were cultured suspended in *a*-MEM containing 10% FBS and plated in 24-well culture plates at a density of 5 × 10^4^ cells per well. The cells were incubated with osteoblasts differentiation medium containing 50 mg/ml of ascorbic acid, 10 mM of *ß*-glycerophosphate, and 10 nM of dexamethasone. Cultured for 21 days, cells were washed with ice-cold PBS and fixed with 4% formalin in PBS for 15 min at 25°C. Then, the cells were washed twice with dH_2_O and stained with 0.1% AR-S-Tris-HCl (pH 4.2) for 30 min at room temperature. The mineralized nodules were observed under an inverted light microscope, and the mineralized nodules were dissolved with 10% acetyl chloride in 10 mM sodium phosphate, followed by the absorbance measurement at 562 nm using a multiplate reader (Zhang et al., 2019).

### Western Blotting

MC3T3-E1 cells were plated in 6-well plates at a density of 1 × 10^5^ cells per well. After overnight incubation, the cells were maintained mineralizing medium and cultured with catalpol (1 and 10 μM). After 2 h, the cells were subjected to high glucose (25.5 mM) for 5 days. The cells were collected using a rubber scraper and incubated for 30 min at 4°C with shaking and centrifuged (10,000 rpm, 15 min, 4°C), and the supernatants were collected. The protein concentration was determined by a BCA protein assay kit (Jiancheng, Nanjing, China). The proteins separated through electrophoresis were transferred onto polyvinylidene difluoride (PVDF) membranes. Afterward, membranes were incubated with blocking buffer (5% (v/v) skimmed milk in 0.05% (v/v) PBS/Tween) for 1 h at RT and, respectively, incubated overnight at 4°C with Runx2, type I collagen, osteocalcin, OPG, RANKL, and mouse GAPDH antibodies. The primary antibodies were detected using alkaline phosphatase-conjugated immunoglobulin G (IgG; Bioworld Technology, St. Louis, United States) at room temperature for 1 h. Membranes were developed colorimetrically by BCIP/NBT alkaline phosphatase color development kit (Beyotime Institute of Biotechnology, Nantong, China). The band density was quantified using ImageJ soft (LI-COR Biosciences) (Lu et al., 2018).

### Cell Migration Assay

For migration assay, the wound-healing assay was done. MC3T3-E1 cells were plated in 12-well plates at a density of 1 × 10^5^ cells per well and incubated for 24 h. The monolayer was then scratched with pipette tips and washed with PBS. Subsequently, the cells were then cultured with catalpol (1 and 10 μM). After 2 h, the cells were subjected to high glucose (25.5 mM) for 24 h according to references and results of pretests. Slides were observed and photographed with a Leica DM LB microscope (Leica Mikrosysteme Vertrieb, Wetzlar, Germany) (Schwarting et al., 2015). The transwell cell migration assay was performed by using a Boyden chamber (polycarbonate membrane with 8-µm pores in 12-well plates). MC3T3-E1 cells were plated onto the upper chamber in 12-well plates at a density of 1 × 10^5^ cells per well in *a*-MEM containing 0.3% FBS, and the lower chambers were filled with 10% FBS medium. The cells were then cultured with catalpol (1 and 10 μM), added to the upper chamber. After 2 h, the cells were subjected to high glucose (25.5 mM) for 24 h. After 24 h of incubation at 37°C, the cells on the upper surface of the membrane were mechanically removed, and the cells adherent to the underside of the transwell membrane were fixed with 4% paraformaldehyde and stained with 4′,6-diamidino-2-phenylindole solution (Kawabata et al., 2018).

### Enzyme-Linked Immumosorbent Assay

After mice were sacrificed, serum was immediately harvested. The concentrations of TRACP and ALP were measured *via* ELISA (Jiancheng Bioengineering Institute, Nanjing, China) kits according to the manufactures’ guidelines and read at 450 nm using a microplate reader (Bio-Rad).

### Microcomputed Tomography (Micro-CT) Scanning

For analysis of bone microstructure and mineralization, the left femurs were subjected to scanning using micro-CT (GE Healthcare, United States). After removing the soft tissues, the left femurs were placed with gauze in the sample holder and were scanned using micro-CT using a resolution of 6 μm, 80 kV, 80 μA, 400 number of views to obtain 3-dimensional images. The eXplore Reconstruction Utility software was used for three-dimensional reconstruction. Trabecular morphometry was characterized by measuring the volumetric parameters of bone mineral density (BMD), bone volume fraction (BVF), BS/BV, trabecular thickness (Tb.Th), trabecular number (Tb.N), and decreases in trabecular spacing (Tb.Sp) (Lontos et al., 2018).

### ALP and TRACP Staining of the Femur and Vertebrae of Mice

The sections of the femur, thoracic vertebra, and lumbar vertebra were stained histochemically for ALP and TRACP activity as an OB and OC marker. The femurs were shortly washed with cold PBS, fixed in 4% paraformaldehyde overnight, and decalcified in 19% EDTA for 7 days. Then, the femur, thoracic vertebra, and lumbar vertebra were dehydrated in an alcohol series and embedded in paraffin and, respectively, cut into serial sections of 10 μm (Li et al., 2017). Respectively, the ALP and TRACP staining were, respectively, performed in accordance with the kit instructions (D001-2-1, D023-1-1, Nanjing Jiancheng Bio, Nanjing, China).

### Data and Statistical Analysis

Data are expressed as the mean ± SD of at least three independent experiments. One-way analysis of variance followed by Dunnett’s *t*-test was used for the statistical analysis (SPSS 13.0 software; SPSS, Chicago, United States). A *p* value of < 0.05 was conventionally considered to be statistically significant. Graphs were drawn using GraphPad Prism (version 6.0 for Windows).

## Results

### The Effect of Catalpol on Structural Parameters of Bone of Diabetic Osteoporosis Mice

**FIGURE 1:**
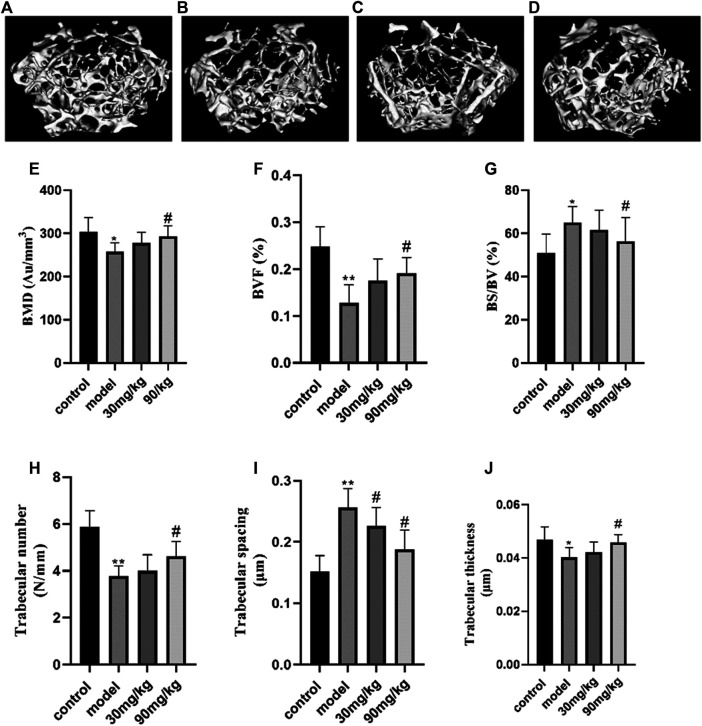
Effect of catalpol on structural parameters of trabecular bone of diabetic osteoporosis mice. Representative 3D reconstructed images analyzed by micro-CT **(A, B, C, D)**. **(A)** Control group; **(B)** model group (diabetic osteoporosis group); **(C)** 30 mg/kg (diabetic osteoporosis group with 30 mg/kg catalpol); **(D)** 90 mg/kg (diabetic osteoporosis group with 90 mg/kg catalpol). The reconstructed images suggested that catalpol could observably inhibit the trabecular bone mass loss and significantly attenuated the degeneration of trabecular bone microarchitecture. **(E)** The effect of catalpol on trabecular BMD of the femoral bone. The trabecular BMD of the femoral bone of the model group significantly decreased (*p* < 0.05), and catalpol (90 mg/kg catalpol) inhibited this effect. The effect of catalpol on BMD BVF, BS/BV, Tb.Th, Tb.N, and Tb. Sp of the mice femur mice in four groups. **(F)** BVF; **(G)** BS/BV(%); **(H)** trabecular number (N/mm); **(I)** trabecular spacing (μm); **(J)** trabecular thickness (μm). The data were expressed as the mean ± SD. **p < 0.01 and *p < 0.05 vs. control and ^#^ *p* < 0.05 vs. model.

We established a diabetic osteoporosis mice model by 55% high-fat diet fed and injecting streptozotocin. Bone mass and bone formation were obviously decreased in osteoporotic femur, compared to normal group mice. The microarchitecture of the distal femur was analyzed by micro-CT and the corresponding bone morphometric parameters shown in Figure 1. The observed degeneration in trabecular bone structure in the model group compared with the normal group and the degeneration of femurs were clearly inhibited by the treatment with catalpol. Histograms represent the parameters of three-dimensional trabecular structural of the distal femur: BMD, BVF, BS/BV, Tb.Th, Tb.N, and Tb. Sp. Compared with the normal group, a high-fat diet and streptozotocin treatment deteriorated the trabecular microarchitecture of the femur, which was demonstrated by decreased BMD (*p* < 0.05), BVF (*p* < 0.01), Tb.Th (*p* < 0.05), and Tb.N (*p* < 0.01) as well as increased Tb. Sp (*p* < 0.01) and BS/BV (*p* < 0.01). Administration of catalpol (90 mg) significantly improved the deteriorated trabecular of the femur. Compared with the model group, the parameters of BV/TV, Tb.N, Tb.Th, and trabecular BMD were significantly enhanced by catalpol, while Tb. Sp, and BS/BV were significantly decreased by catalpol (*p* < 0.05).

### The Effect of Catalpol on Bone Metabolism

**FIGURE 2:**
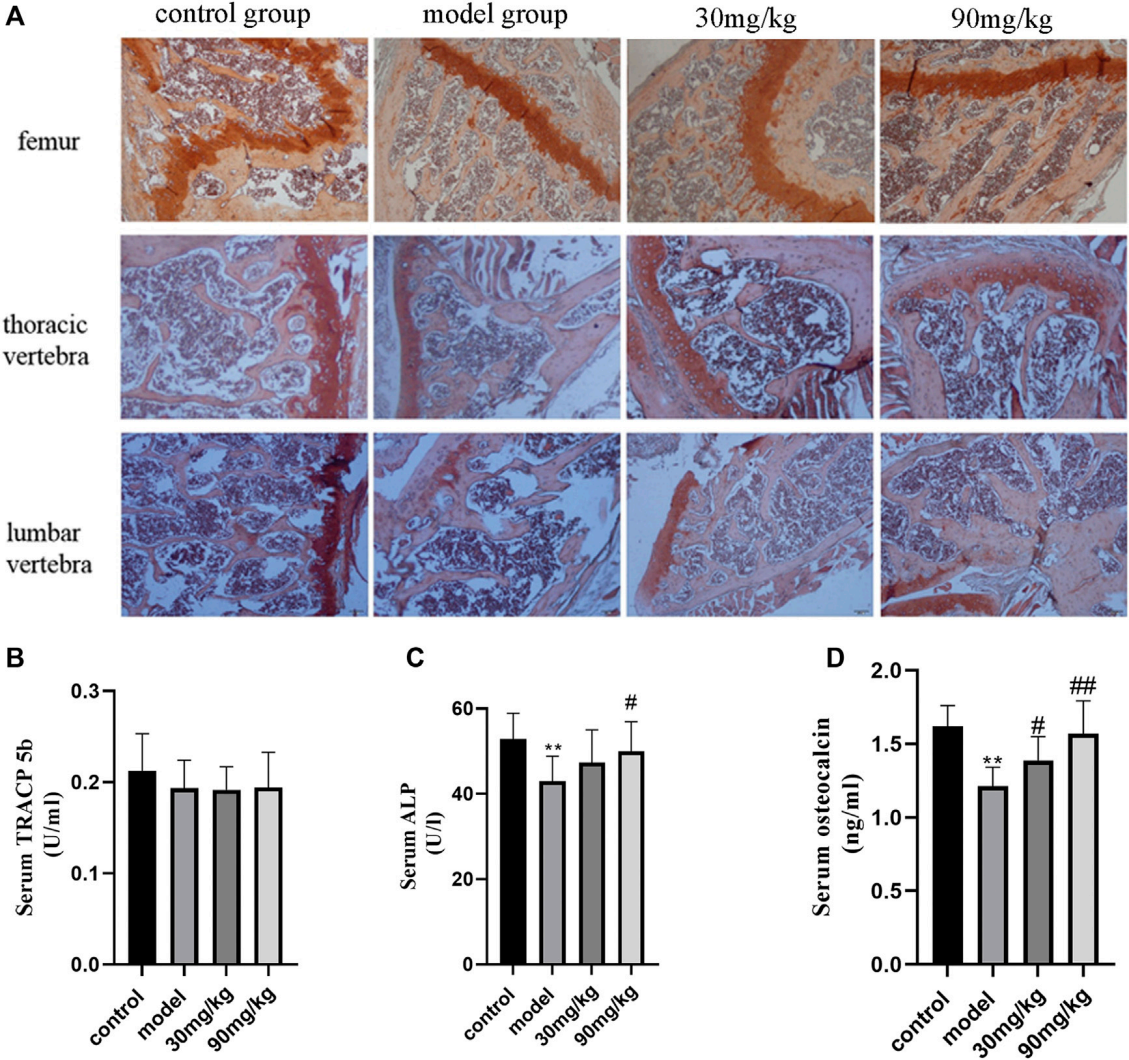
Effect of catalpol on bone formation and bone resorption. **(A)** Representative images of ALP staining. Compared with control group, the levels of ALP in the femur, thoracic vertebra, and lumbar vertebra of diabetic mice were reduced significantly, and catalpol can reverse this effect. **(B)** Serum TRACP-5b levels of mice in four groups assayed by ELISA; there is no significant difference among groups. **(C)** Serum ALP levels of mice in four groups assayed by ELISA. Serum ALP levels of diabetic mice in the model group were decreased significantly, and administration of catalpol (90 mg/kg) significantly improved the ALP levels. **(D)** Serum osteocalcin levels of mice in four groups assayed by ELISA. In the model group, osteocalcin levels were decreased significantly, and administration of catalpol can reverse this decrease. The data were expressed as the mean ± SD. ^**^ *p* < 0.01 vs. control and ^##^ *p* < 0.01 and ^#^ *p* < 0.05 vs. model.

**FIGURE 3:**
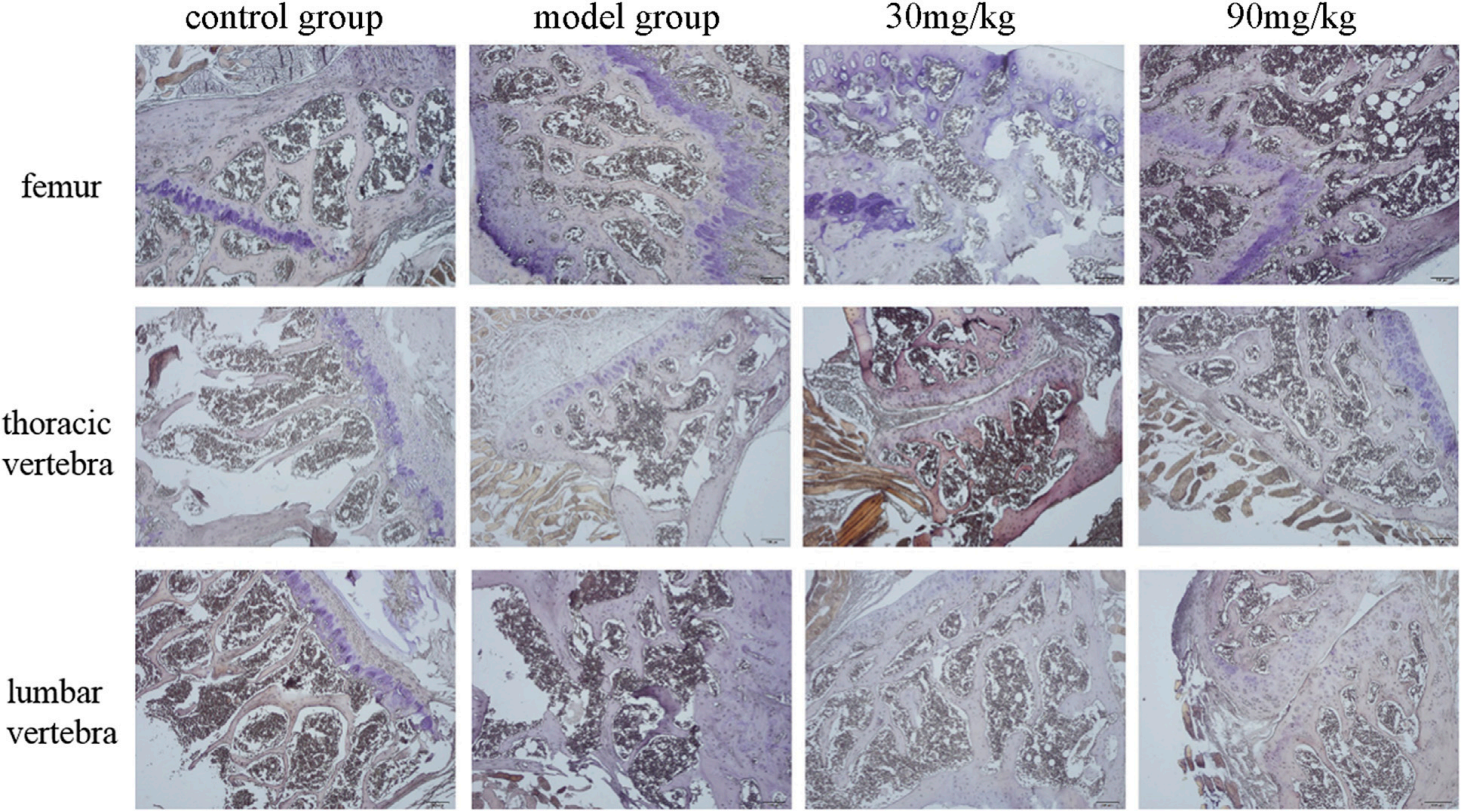
Representative images of TRACP staining.

To assess the role of catalpol on bone formation and bone resorption of the bone of diabetic osteoporosis mice, the serum levels of bone metabolism markers, and the levels of ALP (Figure 2) and TRACP (Figure 3) in the femur, thoracic vertebra, and lumbar vertebra were assayed by ELISA and ALP/TRACP staining. The levels of ALP in femur, thoracic vertebra, and lumbar vertebra of diabetic mice were reduced significantly compared to the control group. Red color shows the ALP in the femur, thoracic vertebra, and lumbar vertebra with significantly elevated levels in catalpol treated groups. This provides further evidence that catalpol stimulates bone formation in different bone tissues of diabetic osteoporosis mice. In addition, serum ALP and osteocalcin levels of diabetic mice in the model group were decreased significantly (*p* < 0.05), and administration of catalpol (90 mg) significantly improved the ALP and osteocalcin levels. These results illustrated that catalpol promoted bone osteoblast differentiation and bone formation *in vitro* and *in vivo*. However, the TRACP levels in the femur, thoracic vertebra, and lumbar vertebra, as well as in serum of diabetic mice, had no significant differences among groups. Catalpol did not show inhibitory effects on bone resorption.

#### The Effect of Catalpol on Osteogenic Differentiation and Calcification of MC3T3-E1 Cells Treated by High Glucose

**FIGURE 4:**
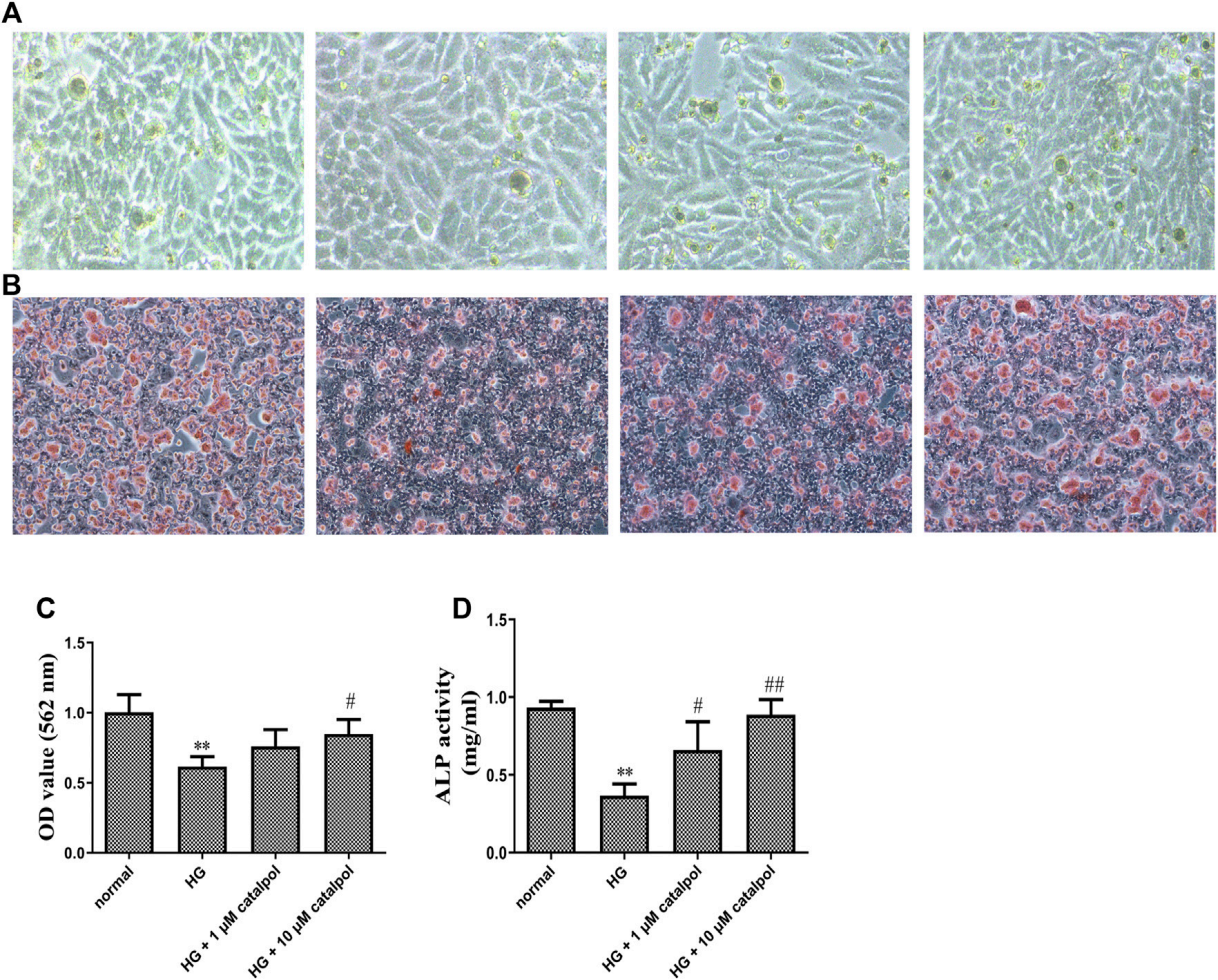
Effect of catalpol on osteogenic differentiation and calcification of MC3T3-E1 cells treated by high glucose. **(A)** Cell differentiation was measured by ALP staining. **(B)** Cell calcification was measured by Alizarin Red staining. **(C)** Catalpol reversed cell calcification according to microplate reader of Alizarin Red staining measured at 562 nm. **(D)** Catalpol increased cell ALP activity treated with high glucose. The data were expressed as the mean ± SD. **p < 0.01 vs. control and ^##^ *p* < 0.01, and ^#^ *p* < 0.05 vs. model.

The preosteoblastic MC3T3-E1 cells were induced to differentiate with an osteogenic medium and the effect of catalpol on cell differentiation was evaluated. The presence of high glucose markedly reduced the osteogenesis potential of MC3T3-E1 cells, which is evident in ALP activity assay and staining. Treatment of MC3T3-E1 osteoblasts under the environment of high glucose with catalpol 1 and 10 μM during differentiation expressed enhanced levels of ALP (Figure 4). To further probe the positive effects of catalpol stimulation on MC3T3-E1 cells treated by high glucose, we investigated calcification using Alizarin Red staining, and Alizarin Red staining activity was quantified by densitometry at 562 nm at microplate reader. Results from the experiment showed that catalpol had an obvious promoting effect on MC3T3-E1 cells in a high concentration of glucose. Compared with the MC3T3-E1 cells in normal situations, the environment of high glucose had an obvious inhibiting effect on calcification. The catalpol groups (1 and 10 μM) showed much denser and wider presence of stained patches of calcified matrix (Figure 4).

**FIGURE 5:**
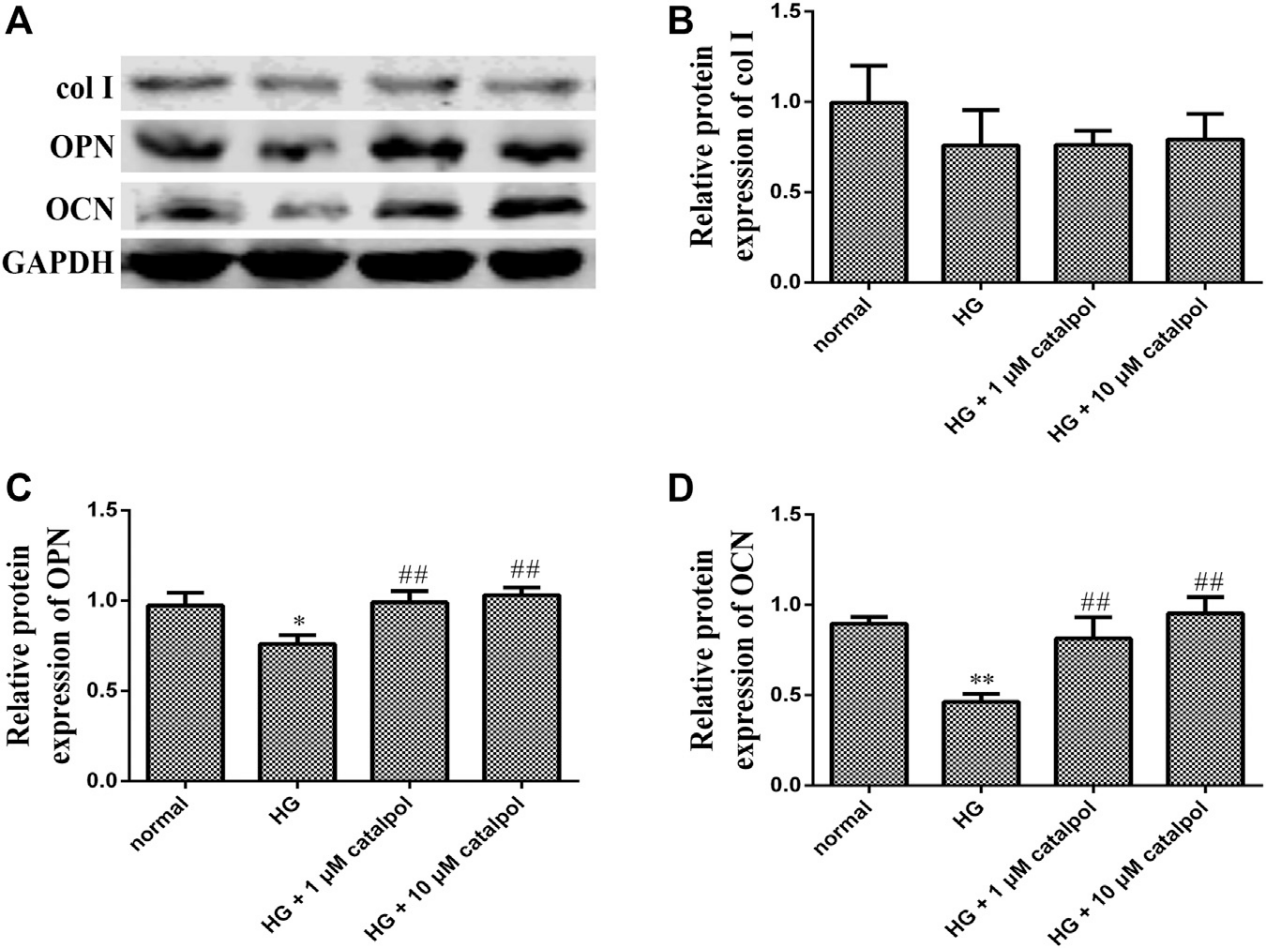
Effect of catalpol on osteogenic differentiation and calcification of MC3T3-E1 cells treated by high glucose. **(A)** Expression of col I, OPN, and OCN related with bone formation was measured by Western blot. Respectively, protein levels were statistically evaluated in column **(B, C, D)**. The data were expressed as the mean ± SD. **p < 0.01 and *p < 0.05 vs. control and ^##^ *p* < 0.01 vs. model.

**FIGURE 6:**
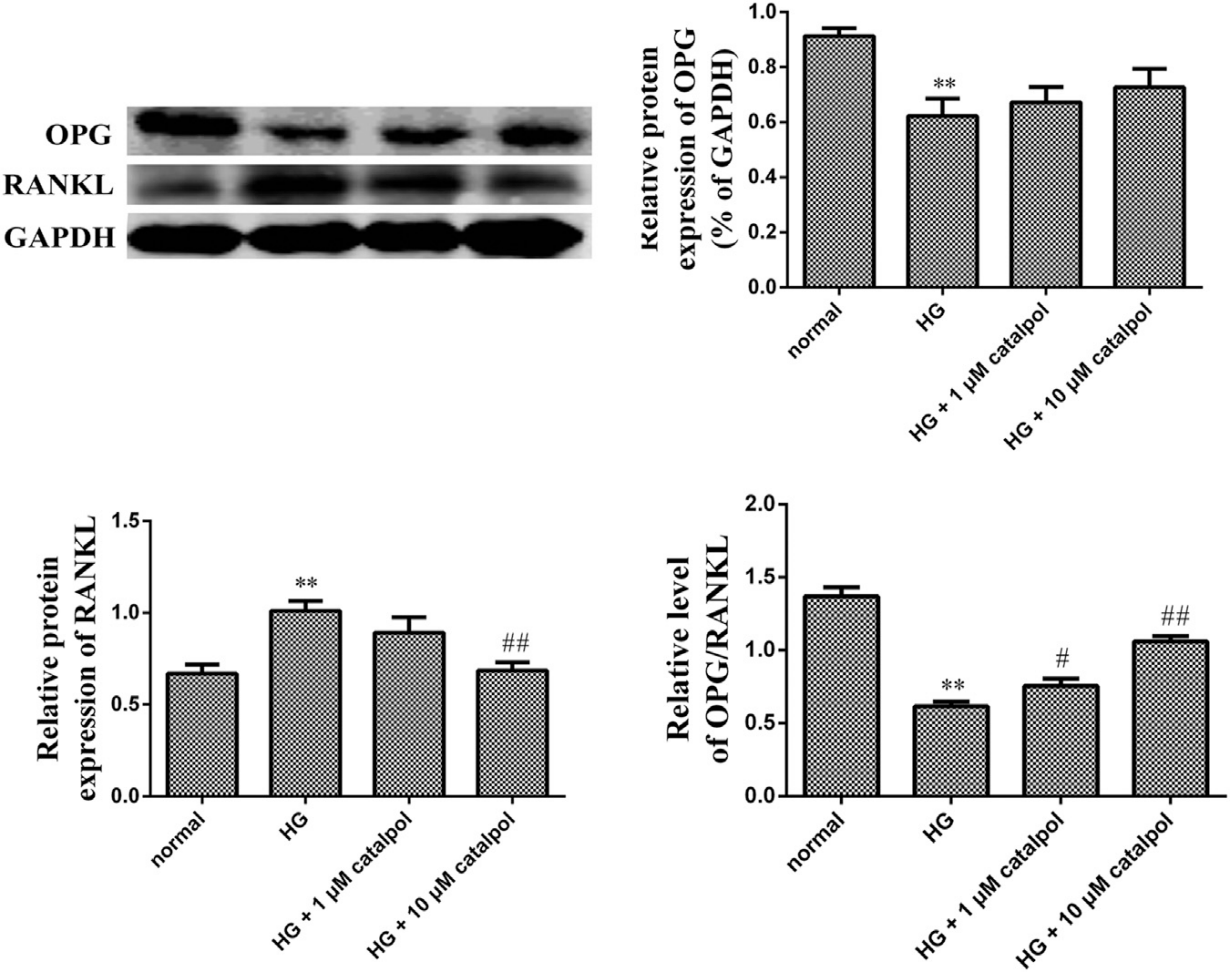
Effect of catalpol on osteogenic differentiation and calcification of MC3T3-E1 cells treated by high glucose. Expression of OPG and RANKL levels was measured during catalpol treatment under high glucose condition by Western blot. Quantitated levels of OPG, RANKL, and the ratio of two proteins were statistically evaluated. The data were expressed as the mean ± SD. **p < 0.01 vs. control and ^##^ *p* < 0.01 and ^#^ *p* < 0.05 vs. model.

To determine the effect of catalpol on osteoblastic cell differentiation, the protein levels involved in the formation of the cellular matrix including middle stage (collagen type 1 and osteopontin) and late stage (osteocalcin) were evaluated by Western blotting after treatment with catalpol. We compared the differences in the levels of osteopontin, type I collagen, and osteocalcin in the normal control group, high-glucose group, and catalpol groups. Treatment with high glucose markedly decreased protein levels of osteopontin and osteocalcin. However, treatment with catalpol 1 and catalpol 10 μM which markedly attenuated this high glucose mediated the inhibition of bone formation. The environment of high glucose reduced the level of type I collagen, but the difference between the normal control group and the high-glucose group was not statistically significant (Figure 5). In addition, high glucose reduced OPG protein level but increased RANKL protein level. Although the level of OPG protein in catalpol groups had little change compared with high-glucose groups, catalpol reduced the RANKL protein level and increased the ratio of OPG/RANKL significantly (Figure 6).

### The Effect of Catalpol on Migration of MC3T3-E1 Cells Treated by High Glucose

**FIGURE 7:**
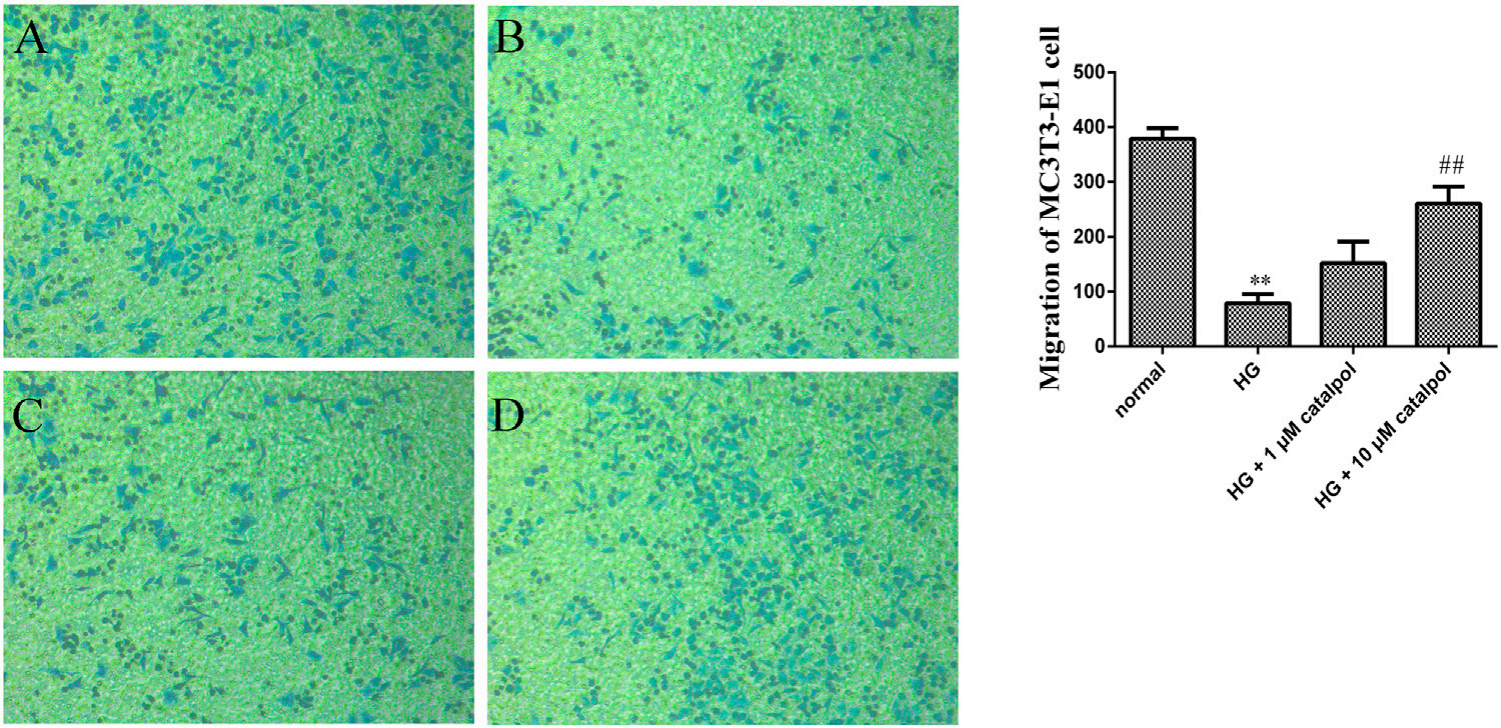
Effect of catalpol on migration of MC3T3-E1 cells treated by high glucose. Cell invasive capacity was measured by the transwell cell migration assay. The level of migration was statistically evaluated, as shown in the column. **(A)** Normal group. **(B)** HG group. **(C)** HG + 1 μM catalpol. **(D)** HG + 10 μM catalpol. The data were expressed as the mean ± SD. **p < 0.01 vs. control and ^##^ *p* < 0.01 vs. model.

**FIGURE 8:**
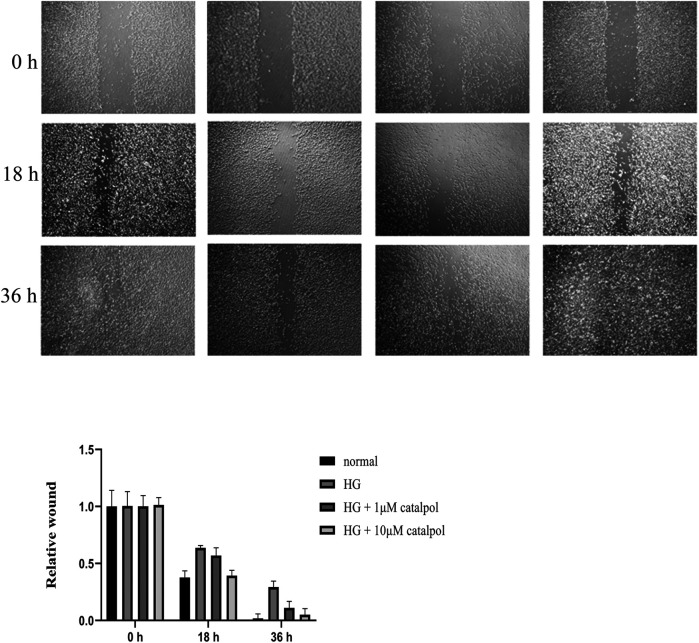
Effect of catalpol on migration of MC3T3-E1 cells treated by high glucose. Cell migration was measured by the wound-healing assay. The level of migration was statistically evaluated, as shown in the column. The data were expressed as the mean ± SD. **p < 0.01 vs. control and ^##^ *p* < 0.01 vs. model.

In the process of bone regeneration, osteoblast precursors and their progeny are required to migrate within the three-dimensional bone space (Xie et al., 2018). Therefore, inducing the migration of osteoblast might play a role in osteoporosis. To investigate the effects of catalpol on the migration capacity of osteoblasts, a transwell assay was performed. As shown in Figure 7, MC3T3-E1 cells treated with high glucose have lower migration behavior compared with the normal group. Catalpol (1 and 10 μM) significantly enhanced the migration behavior of MC3T3-E1 cells treated by high glucose after 24 h of culture. In addition, cell migration activity was also assessed using the wound-healing assay. In Figure 8, compared with the MC3T3-E1 cells in normal situations, the environment of high glucose had an obvious inhibiting effect on cell migration activity. Catalpol enhanced cell motility and scattering following gap formation in MC3T3-E1 cells treated by high glucose. Collectively, these findings from the transwell cell migration assay and the wound-healing assay demonstrate that catalpol promotes migration of MC3T3-E1 cells treated by high glucose.

## Discussion

Recent studies have shown that patients with type 1 DM (T1DM) and T2DM have an increased risk of osteoporotic fractures and bone fragility induced by diabetes mellitus. Diabetic osteoporosis has been recently recognized as one of the important complications. As diabetic osteoporosis is more complex than other types of osteoporosis, so too are its etiology and pathology. The reduction in bone mineral density and deterioration of the bone quality induced by prolonged hyperglycemia is the main reason for the rising risk of osteoporotic fracture in patients with diabetes (Bai et al., 2019). Up to now, numerous researches have shown that suppression of bone formation is the main cause of higher vertebral and overall fracture rates in diabetes patients. However, bone resorption and osteoclast activity in diabetes assumed to have little to no influence on deterioration of the bone architecture compared with decreased bone formation. In addition, the OPG/RANKL ratio is significantly lower in osteoblasts grown in high glucose and treatment with catalpol increases the OPG/RANKL ratio. These results indicate that catalpol has the potential to inhibit bone resorption via the OPG/RANKL pathway. However, there is nearly no difference in bone resorption between catalpol group mice and diabetic mice. The possible reason is that the level of RANKL reflects the changes in systemic physiology including bone, vascular tissue, and other tissues (Liu et al., 2019). More bone resorption markers should be taken into analysis in future studies.

Bone is a specialized and dynamic tissue which is continuously remodeled by the coordinated actions of osteoclast and osteoblast. Throughout the dynamic process of bone mobilization, osteoclasts resorb mineralized bone and form bone lacuna; thereby osteoblasts embed into the lacuna and differentiate into osteocytes (Bai et al., 2019). When the dynamic balance between bone resorption by osteoclasts and bone formation by osteoblasts is disrupted in diabetic osteoporosis, it leads to increasing the risk of fragility fracture. The hyperglycemic conditions induce the accelerated formation and accumulation of AGEs, which directly inhibits osteoblasts and upregulates sclerostin in osteocytes. In addition, the high-glucose level increases the secretion levels of sclerostin in osteocytes and further inhibits osteoblasts by suppressing the Wnt/β-catenin pathway (Ye et al., 2018). In this study, type 2 diabetic mice showed significant inhibition of bone formation, and high glucose significantly inhibited the differentiation and migration of MC3T3-E1 cells. However, there was no significant change in bone resorption compared with normal mice. In the process of bone remodeling from resorption to formation, high glucose main influenced reversal phase and formation and mineralization phase in which the proliferation, differentiation, and migration of osteoblasts play an important role (Carbone et al., 2017).

The roots of *Rehmannia glutinosa* (Dihuang in Chinese) have been widely used in combination with other TCM herbs to treat patients with diabetes, diabetic complications, and osteoporosis. Recent meta-analyses based on 107 TCM clinical trials have shown that the prescriptions contained *Rehmanniae Radix* has promising effects on alleviating bone loss and reducing pain of osteoporotic patients (Pierce and Waite, 1987). Catalpol, the principal effective component of *Rehmannia Radix*, exhibits various pharmacological activities, especially potent antioxidant, antitumor, antiosteoporosis, and antidiabetic effects. In addition, catalpol is the only isolated effective compound of *Rehmannia Radix that* has bone preventive effect *in vitro* and *in vivo* (Qian et al., 2015; Sun et al., 2018; Terpos et al., 2018).

In general, bone remodeling is controlled by the balance between bone formation and bone resorption. After the first phase of osteoclastic bone resorption, preosteoblast migrates into the resorption cavities, followed by proliferation and differentiation of osteoblasts (Pajarinen et al., 2019). In this study, we revealed that catalpol showed significant osteogenesis promoting effects in diabetic osteoporosis mice model and high glucose osteoblast model. More efficient promoting osteogenesis drugs are of great clinical value of osteoporotic therapy. Unlike postmenopausal osteoporosis, the levels of bone turnover in patients with T1D and T2D are significantly reduced levels compared with controls (Hu et al., 2016b). In addition, hyperglycemia induces higher serum levels of sclerostin, a bone-signaling peptide secreted from the osteocyte, and inhibits osteoblast activity by blocking the noncanonical Wnt signaling pathways. Therefore, activating the Wnt signaling inhibited by redundant sclerostin and accelerating osteoblast to further differentiation and maturation have positive effects on bone loss in diabetic osteoporosis. The effects of catalpol on bone formation and calcium deposition in BMSCs *in vitro* was partly via activation of upregulation of Wnt/β-catenin pathway (Ning et al., 2018). There are several studies explaining the mechanism of catalpol on osteoblast. KDM7A are key target of catalpol on the proliferation and differentiation of osteoblasts induced by high glucose (Cheng et al., 2020). There is also one study proving that catalpol enhanced bone formation by regulating IGF-1/PI3K/mTOR signaling pathways in osteoblast (Gong et al., 2019). In the present study, we observed that the bone protection on osteoporosis of diabetic mice and osteoblasts under high glucose environment may be involved in various factors and mechanisms. However, there was also one study that was inconsistent with our conclusion. The study found that catalpol protected bone loss by inhibiting osteoclast activity (Xu, 2014). These possibilities should be followed up in future studies.

The inhibition effect of the high glucose environment on osteoblast differentiation may be a consequence of multiple mechanisms, including activating pyroptosis pathway, increasing ROS production (Rezaeepoor et al., 2018), and activating GSK3β (Hu et al., 2016a). However, there are several limitations that need to be addressed in future study. Although the animal model used in this experiment basically reflects the bone degeneration of type 2 diabetes, transgenic mice with type 2 diabetes can more realistically simulate the mechanism of bone loss of diabetic osteoporosis in future study. Besides, the detailed mechanism actions of catalpol need further research to become a candidate drug in the treatment of diabetic osteoporosis.

## Data Availability

The raw data supporting the conclusion of this article will be made available by the authors, without undue reservation.

## References

[B1] Bai Y. Zhu R. Tian Y. Li R. Chen B. Zhang H. (2019). Catalpol in diabetes and its complications: a review of pharmacology, pharmacokinetics, and safety. Molecules. 24 (18), 3302. 10.3390/molecules24183302 PMC6767014 31514313

[B2] Carbone E. J. Rajpura K. Allen B. N. Cheng E. Ulery B. D. Lo K. W. (2017). Orthotropic nanoscale drug delivery systems based on small molecule bone-targeting moieties. Nanomedicine. 13 (1), 37–47. 10.1016/j.nano.2016.08.015 27562211

[B37] Chandran M. (2017). Diabetes drug effects on the skeleton. Calcif. Tissue Int. 100 (2), 133–149. 10.1007/s00223-016-0203-x 27815568

[B3] Cheng J. Xu H. Y. Liu M. M. Cai J. P. Wang L. Hua Z. (2020). Catalpol promotes the proliferation and differentiation of osteoblasts induced by high glucose by inhibiting KDM7A. Diabetes Metab Syndr. Obes. 13, 705–712. 10.2147/DMSO.S246433 32214833 PMC7081648

[B4] Dong K. Hao P. Xu S. Liu S. Zhou W. Yue X. (2017). Alpha-lipoic acid alleviates high-glucose suppressed osteogenic differentiation of mc3t3-E1 cells via antioxidant effect and PI3K/akt signaling pathway. Cell. Physiol. Biochem. 42 (5), 1897–1906. 10.1159/000479605 28772267

[B5] Gong W. Zhang N. Cheng G. Zhang Q. He Y. Shen Y. (2019). Rehmannia glutinosa Libosch extracts prevent bone loss and architectural deterioration and enhance osteoblastic bone formation by regulating the IGF-1/PI3K/mTOR pathway in streptozotocin-induced diabetic rats. Int. J. Mol. Sci. 20 (16), 3964. 10.3390/ijms20163964 PMC6720794 31443143

[B6] Ho-Shui-Ling A. Bolander J. Rustom L. E. Johnson A. W. Luyten F. P. Picart C. (2018). Bone regeneration strategies: engineered scaffolds, bioactive molecules and stem cells current stage and future perspectives. Biomaterials. 180, 143–162. 10.1016/j.biomaterials.2018.07.017 30036727 PMC6710094

[B7] Hu H. Xuan Y. Xue M. Cheng W. Wang Y. Li X. (2016a). Semaphoring 3A attenuates cardiac autonomic disorders and reduces inducible ventricular arrhythmias in rats with experimental myocardial infarction. BMC Cardiovasc. Disord. 16, 16. 10.1186/s12872-016-0192-8 26787044 PMC4719212

[B8] Hu Z. Q. Zhou S. L. Zhou Z. J. Luo C. B. Chen E. B. Zhan H. (2016b). Overexpression of semaphoring 3A promotes tumor progression and predicts poor prognosis in hepatocellular carcinoma after curative resection. Oncotarget. 7 (32), 51733–51746. 10.18632/oncotarget.10104 27351132 PMC5239511

[B9] Jiang P. Xiang L. Chen Z. Lu H. Zhou L. Yang L. (2018). Catalpol alleviates renal damage by improving lipid metabolism in diabetic db/db mice. Am. J. Transl. Res. 10 (6), 1750–1761. 30018716 PMC6038072

[B10] Kanazawa I. Takeno A. Tanaka K. I. Yamane Y. Sugimoto T. (2018). Osteoporosis and vertebral fracture are associated with deterioration of activities of daily living and quality of life in patients with type 2 diabetes mellitus. J. Bone Miner. Metabol. 37.(3):503–511. 10.1007/s00774-018-0948-6 30191456

[B11] Kawabata T. Tokuda H. Sakai G. Fujita K. Matsushima-Nishiwaki R. Kuroyanagi G. (2018). HSP70 inhibitor suppresses IGF-I-stimulated migration of osteoblasts through p44/p42 MAP kinase. Biomedicines. 6 (4), 109. 10.3390/biomedicines6040109 PMC6316248 30469446

[B12] King S. Klineberg I. Levinger I. Brennan-Speranza T. C. (2016). The effect of hyperglycaemia on osseointegration: a review of animal models of diabetes mellitus and titanium implant placement. Arch Osteoporos. 11 (1), 29. 10.1007/s11657-016-0284-1 27637755

[B13] Lai N. Zhang J. Ma X. Wang B. Miao X. Wang Z. (2015). Regulatory effect of catalpol on Th1/Th2 cells in mice with bone loss induced by estrogen deficiency. Am. J. Reprod. Immunol. 74 (6), 487–498. 10.1111/aji.12423 26303620

[B14] Li F. Yang Y. Zhu P. Chen W. Qi D. Shi X. (2012). Echinacoside promotes bone regeneration by increasing OPG/RANKL ratio in MC3T3-E1 cells. Fitoterapia. 83 (8), 1443–1450. 10.1016/j.fitote.2012.08.008 22951288

[B15] Li Z. Hao J. Duan X. Wu N. Zhou Z. Yang F. (2017). The role of semaphorin 3A in bone remodeling. Front. Cell. Neurosci. 11, 40. 10.3389/fncel.2017.00040 28293171 PMC5328970

[B16] Liu C. Ma R. Wang L. Zhu R. Liu H. Guo Y. (2017). Rehmanniae Radix in osteoporosis: a review of traditional Chinese medicinal uses, phytochemistry, pharmacokinetics and pharmacology. J. Ethnopharmacol. 198, 351–362. 10.1016/j.jep.2017.01.021 28111216

[B17] Liu S. Y. Meng X. F. Liu S. W. Hao C. L. Li L. F. Zhang N. (2019). Effect of Bushen Huoxue decoction on inhibiting osteogenic differentiation of vascular smooth cells by regulating OPG/RANK/RANKL system in vascular calcification. Ann. Transl. Med. 7 (6), 125. 10.21037/atm.2019.02.33 31032280 PMC6465438

[B18] Lontos K. Adamik J. Tsagianni A. Galson D. L. Chirgwin J. M. Suvannasankha A. (2018). The role of semaphorin 4D in bone remodeling and cancer metastasis. Front. Endocrinol. 9, 322. 10.3389/fendo.2018.00322 PMC6018527 29971044

[B19] Lu Q. Zhou Y. Hao M. Li C. Wang J. Shu F. (2018). The mTOR promotes oxidative stress-induced apoptosis of mesangial cells in diabetic nephropathy. Mol. Cell. Endocrinol. 473, 31–43. 10.1016/j.mce.2017.12.012 29277549

[B20] Ma R. Zhu R. Wang L. Guo Y. Liu C. Liu H. (2016). Diabetic osteoporosis: a review of its traditional Chinese medicinal use and clinical and preclinical research. Evid. Based Complement Alternat. Med. 2016, 3218313. 10.1155/2016/3218313 27698674 PMC5028800

[B21] Ning L. Li Z. Wei D. Chen H. Yang C. Wu D. (2018). Urinary semaphoring 3A as an early biomarker to predict contrast-induced acute kidney injury in patients undergoing percutaneous coronary intervention. Braz. J. Med. Biol. Res. 51 (4), e6487. 10.1590/1414-431x20176487 29513790 PMC5856432

[B22] Pajarinen J. Lin T. Gibon E. Kohno Y. Maruyama M. Nathan K. (2019). Mesenchymal stem cell-macrophage crosstalk and bone healing. Biomaterials 196, 80–89. 10.1016/j.biomaterials.2017.12.025 29329642 PMC6028312

[B38] Palermo A. D'Onofrio L. Eastell R. Schwartz A. V. Pozzilli P. Napoli N. (2015) Oral anti-diabetic drugs and fracture risk, cut to the bone: safe or dangerous? A narrative review. Osteoporos. Int. 26 (8), 2073–89. 10.1007/s00198-015-3123-0 25910746

[B23] Pereira M. Gohin S. Roux J. P. Fisher A. Cleasby M. E. Mabilleau G. (2017). Eventide improves bone quality in a murine model of genetically inherited type 2 diabetes mellitus. Front. Endocrinol. 8, 327. 10.3389/fendo.2017.00327 PMC5701968 29209277

[B24] Pierce W. M. Jr. Waite L. C. (1987). Bone-targeted carbonic anhydrase inhibitors: effect of a proinhibitor on bone resorption *in vitro* . Proc. Soc. Exp. Biol. Med. 186 (1), 96–102. 10.3181/00379727-186-42590a 3628257

[B25] Qian C. Zhu C. Yu W. Jiang X. Zhang F. (2015). High-fat diet/low-dose streptozotocin-induced type 2 diabetes in rats impacts osteogenesis and Wnt signaling in bone marrow stromal cells. PloS One. 10 (8), e0136390. 10.1371/journal.pone.0136390 26296196 PMC4546646

[B26] Rezaeepoor M. Ganjalikhani-Hakemi M. Shapoori S. Eskandari N. Sharifi M. Etemadifar M. (2018). Semaphoring-3A as an immune modulator is suppressed by MicroRNA-145-5p. Cell J. 20 (1), 113–119. 10.22074/cellj.2018.4842 29308627 PMC5759673

[B27] Schwarting T. Pretzsch S. Debus F. Ruchholtz S. Lechler P. (2015). The effect of cyclooxygenase inhibition on tendon-bone healing in an in vitro coculture model. Mediat. Inflamm. 2015, 926369. 10.1155/2015/926369 PMC4438175 26063979

[B28] Sun J. Wei X. Wang Z. Liu Y. Lu J. Lu Y. (2018). Inflammatory milieu cultivated Sema3A signaling promotes chondrocyte apoptosis in knee osteoarthritis. J. Cell. Biochem. 119 (3), 2891–2899. 10.1002/jcb.26470 29111592

[B29] Terpos E. Ntanasis-Stathopoulos I. Christoulas D. Bagratuni T. Bakogeorgos M. Gavriatopoulou M. (2018). Semaphoring 4D correlates with increased bone resorption, hypercalcemia, and disease stage in newly diagnosed patients with multiple myeloma. Blood Canc. J. 8 (5), 42. 10.1038/s41408-018-0075-6 PMC5945651 29748532

[B30] Vestergaard P. (2007). Discrepancies in bone mineral density and fracture risk in patients with type 1 and type 2 diabetes—a meta-analysis. Osteoporos. Int. 18 (4), 427–444. 10.1007/s00198-006-0253-4 17068657

[B31] Xie H. Wang Q. Zhang X. Wang T. Hu W. Manicum T. (2018). Possible therapeutic potential of berberine in the treatment of STZ plus HFD-induced diabetic osteoporosis. Biomed. Pharmacother. 108, 280–287. 10.1016/j.biopha.2018.08.131 30223099

[B32] Xu R. (2014). Semaphoring 3A: a new player in bone remodeling. Cell Adhes. Migrat. 8 (1), 5–10. 10.4161/cam.27752 PMC3974794 24589620

[B33] Yan J. Wang C. Jin Y. Meng Q. Liu Q. Liu Z. (2018). Catalpol ameliorates hepatic insulin resistance in type 2 diabetes through acting on AMPK/NOX4/PI3K/AKT pathway. Pharmacol. Res. 130, 466–480. 10.1016/j.phrs.2017.12.026 29284152

[B34] Ye W. L. Zhao Y. P. Cheng Y. Liu D. Z. Cui H. Liu M. (2018). Bone metastasis target redox-responsive micell for the treatment of lung cancer bone metastasis and anti-bone resorption. Artif Cells Nanomed Biotechnol. 46 (Suppl. 1), 380–391. 10.1080/21691401.2018.1426007 29336169

[B35] Zhang Q. Zhao L. Shen Y. He Y. Cheng G. Yin M. (2019). Curculigoside protects against excess-iron-induced bone loss by attenuating akt-FoxO1-dependent oxidative damage to mice and osteoblastic MC3T3-E1 cells. Oxid. Med. Cell Longev. 2019, 9281481. 10.1155/2019/9281481 31949885 PMC6948300

[B36] Zhou F. Mei J. Han X. Li H. Yang S. Wang M. (2019). Kinsenoside attenuates osteoarthritis by repolarizing macrophages through inactivating NF-κB/MAPK signaling and protecting chondrocyte. Acta Pharm. Sin. B. 9 (5), 973–985. 10.1016/j.apsb.2019.01.015 31649847 PMC6804452

