# Supplementary figures and images for: Catalpol Protects Against High Glucose-Induced Bone Loss by Regulating Osteoblast Function
Source: Front Pharmacol. 2021 Mar 10;12:626621. doi: 10.3389/fphar.2021.626621 (PMC7987667; doi:10.3389/fphar.2021.626621)

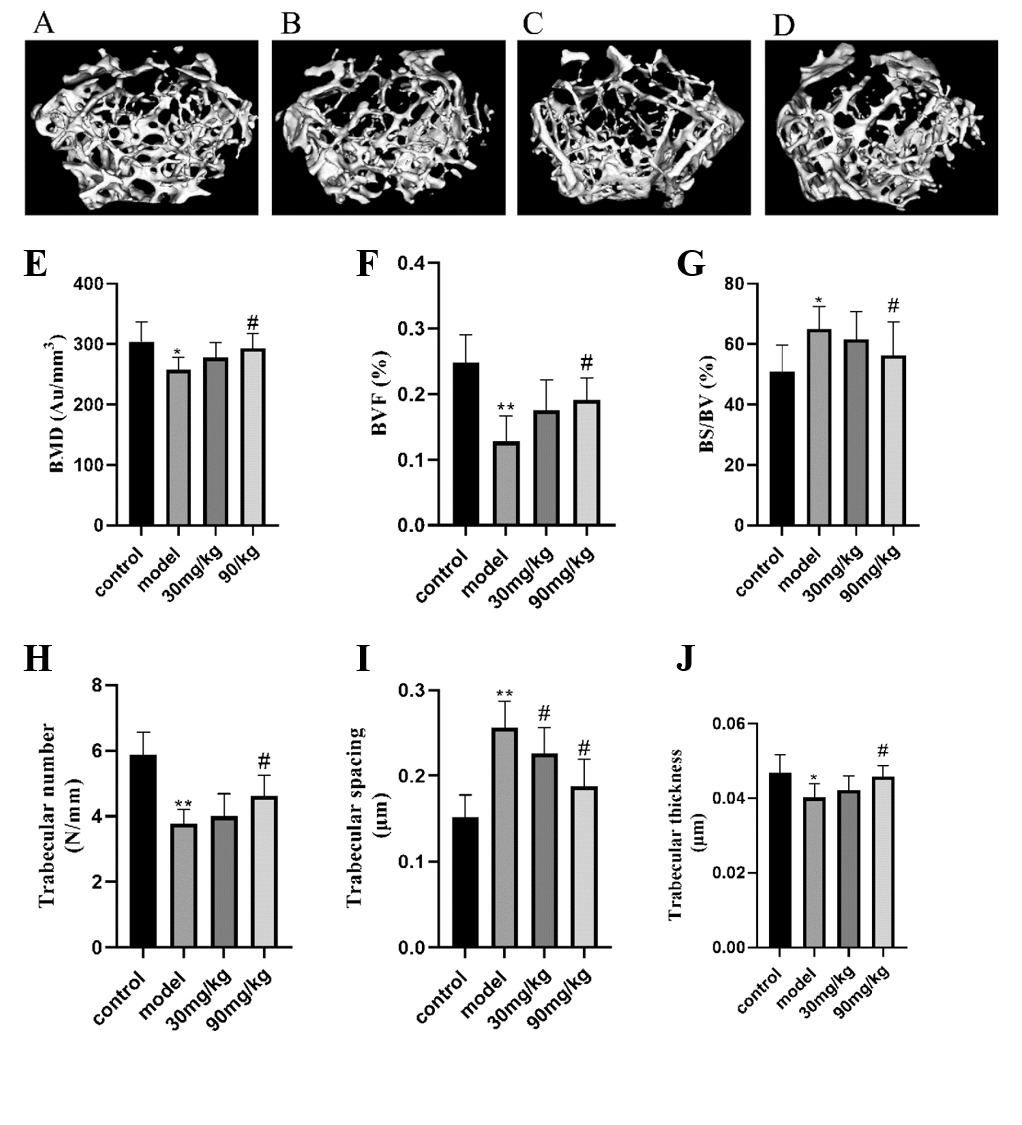

Supplement: Supplementary file 1 [file datasheet1.zip › Supplementary Material Presentation/Figure 1.tif]

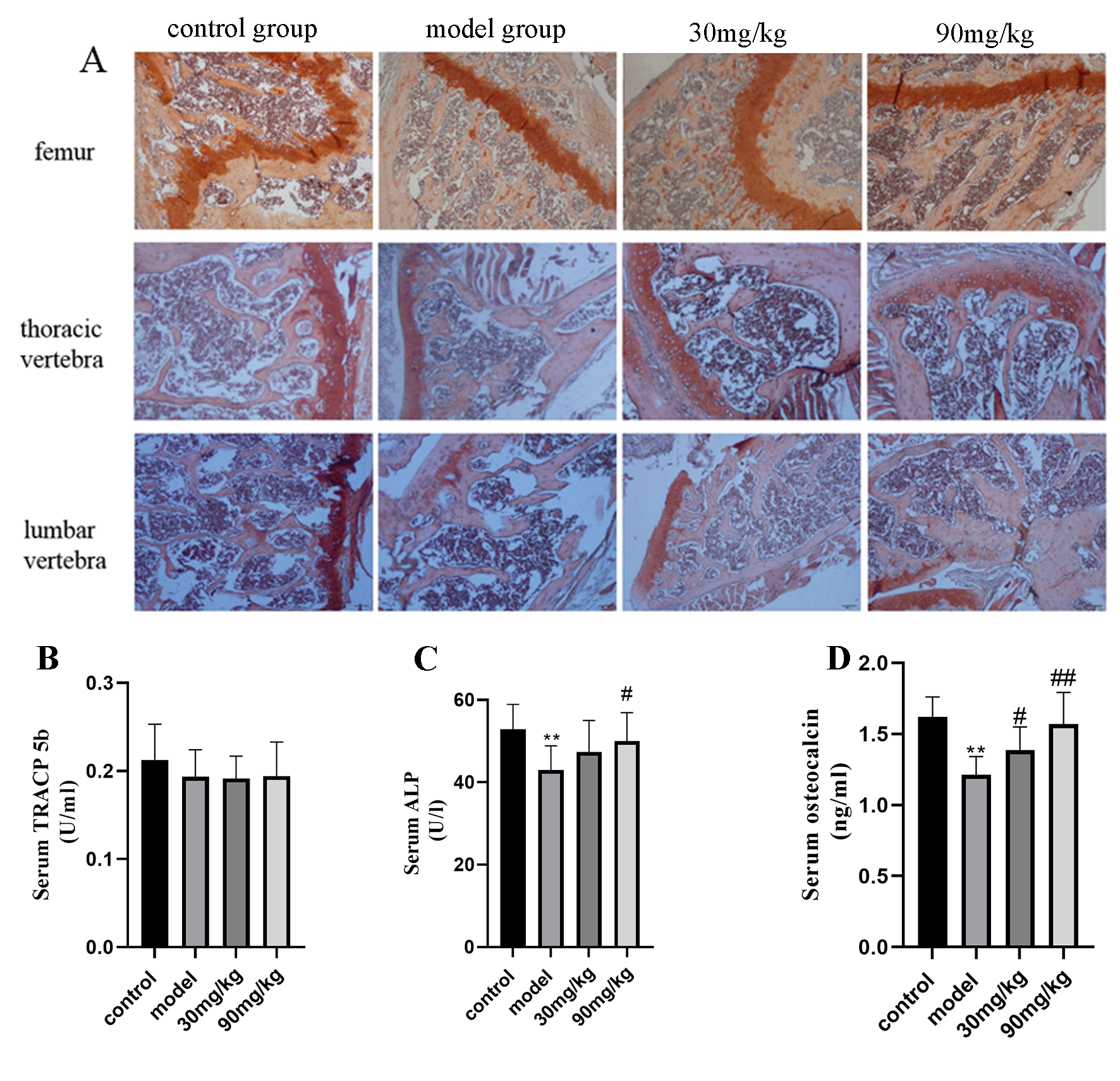

Supplement: Supplementary file 1 [file datasheet1.zip › Supplementary Material Presentation/Figure 2.tif]

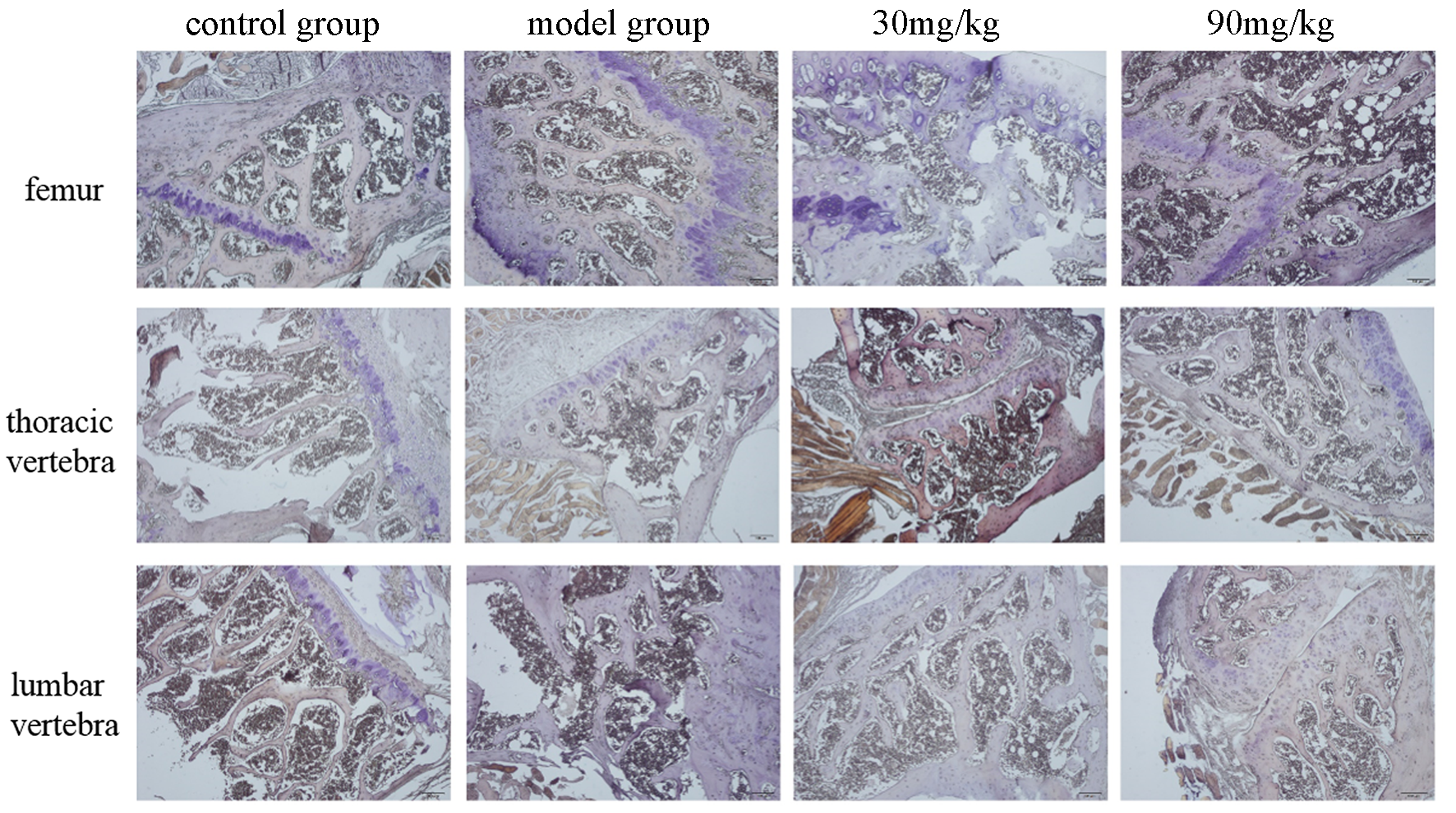

Supplement: Supplementary file 1 [file datasheet1.zip › Supplementary Material Presentation/Figure 3.tif]

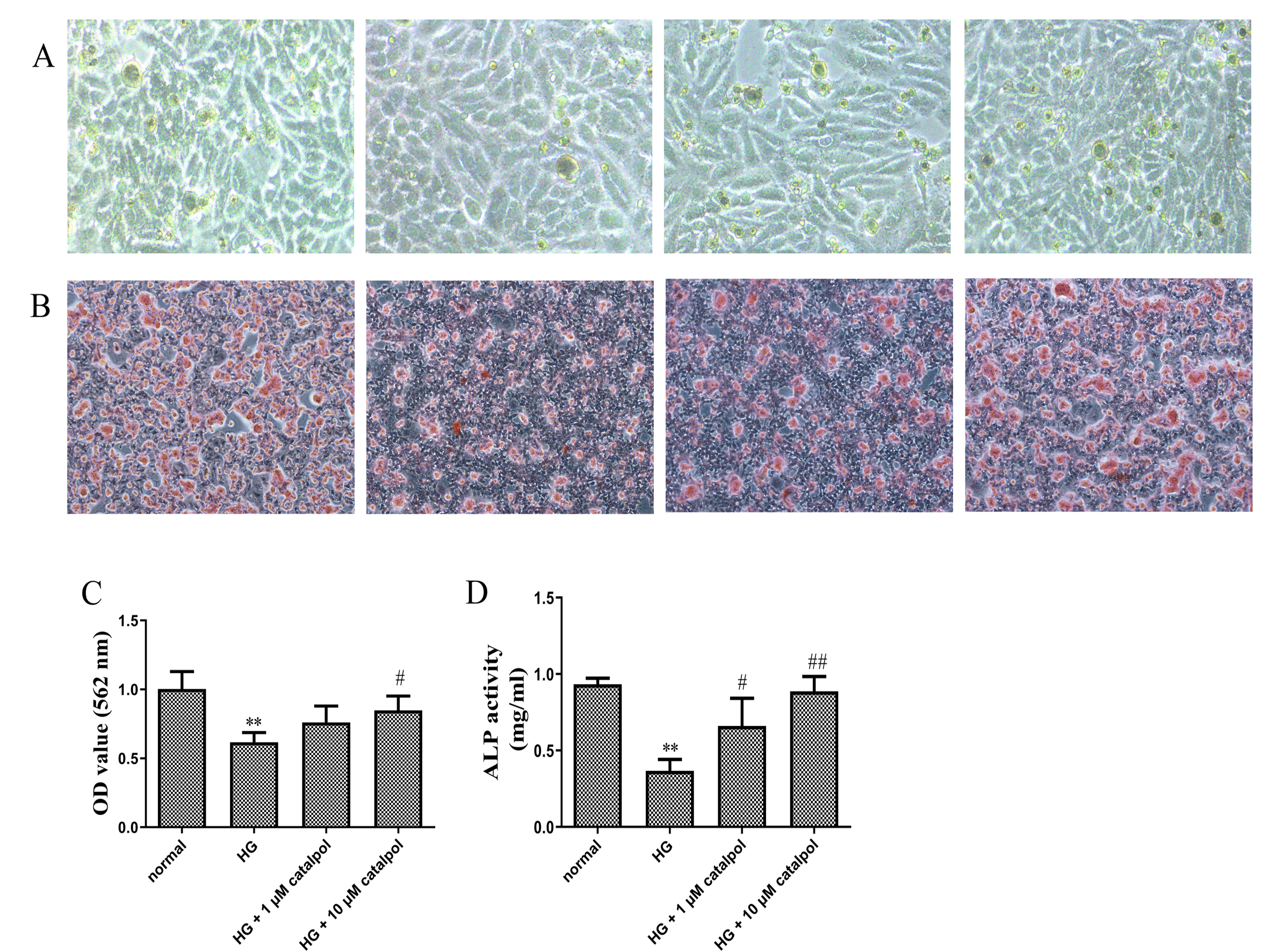

Supplement: Supplementary file 1 [file datasheet1.zip › Supplementary Material Presentation/Figure 4.tif]

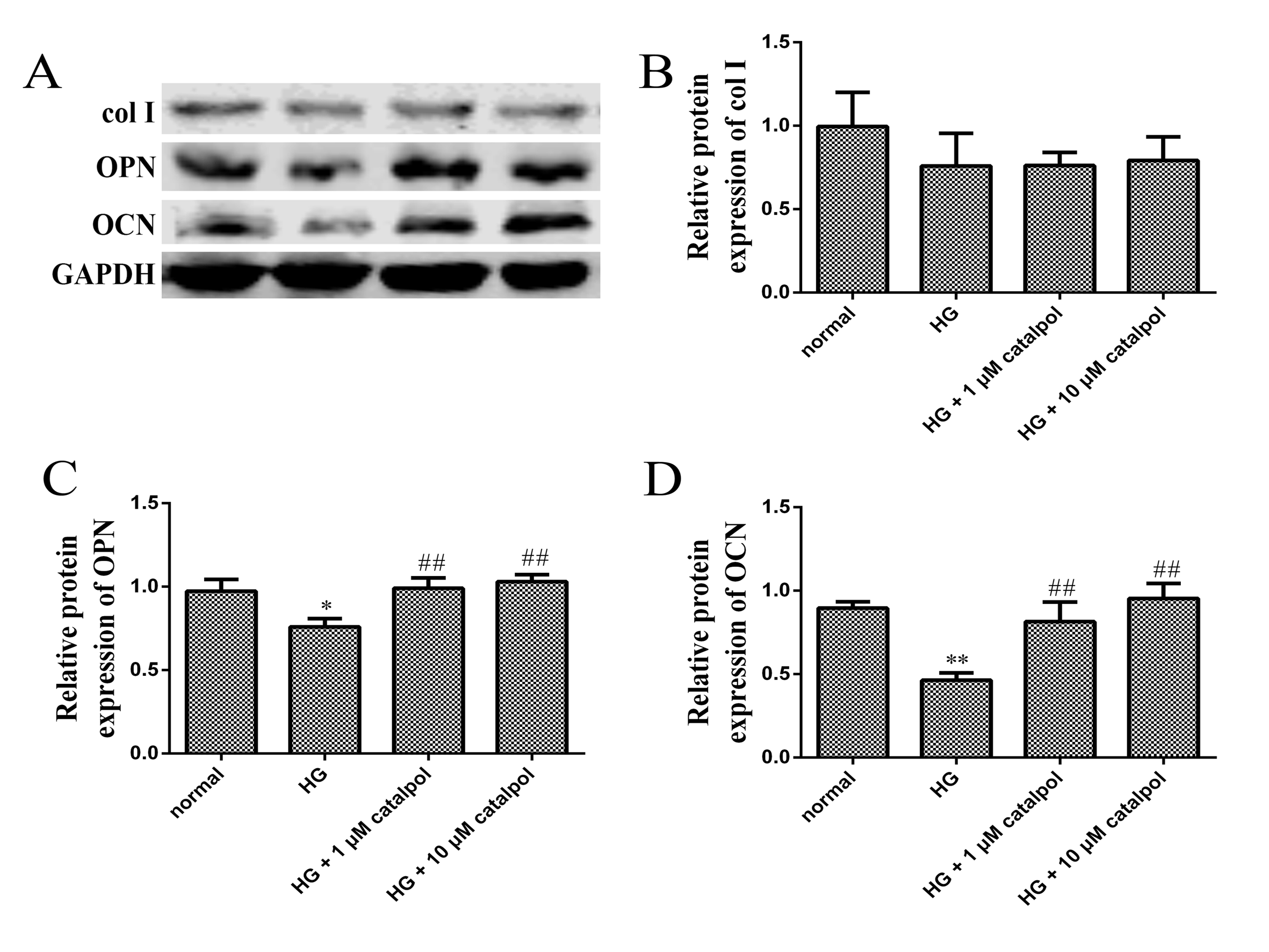

Supplement: Supplementary file 1 [file datasheet1.zip › Supplementary Material Presentation/Figure 5.tif]

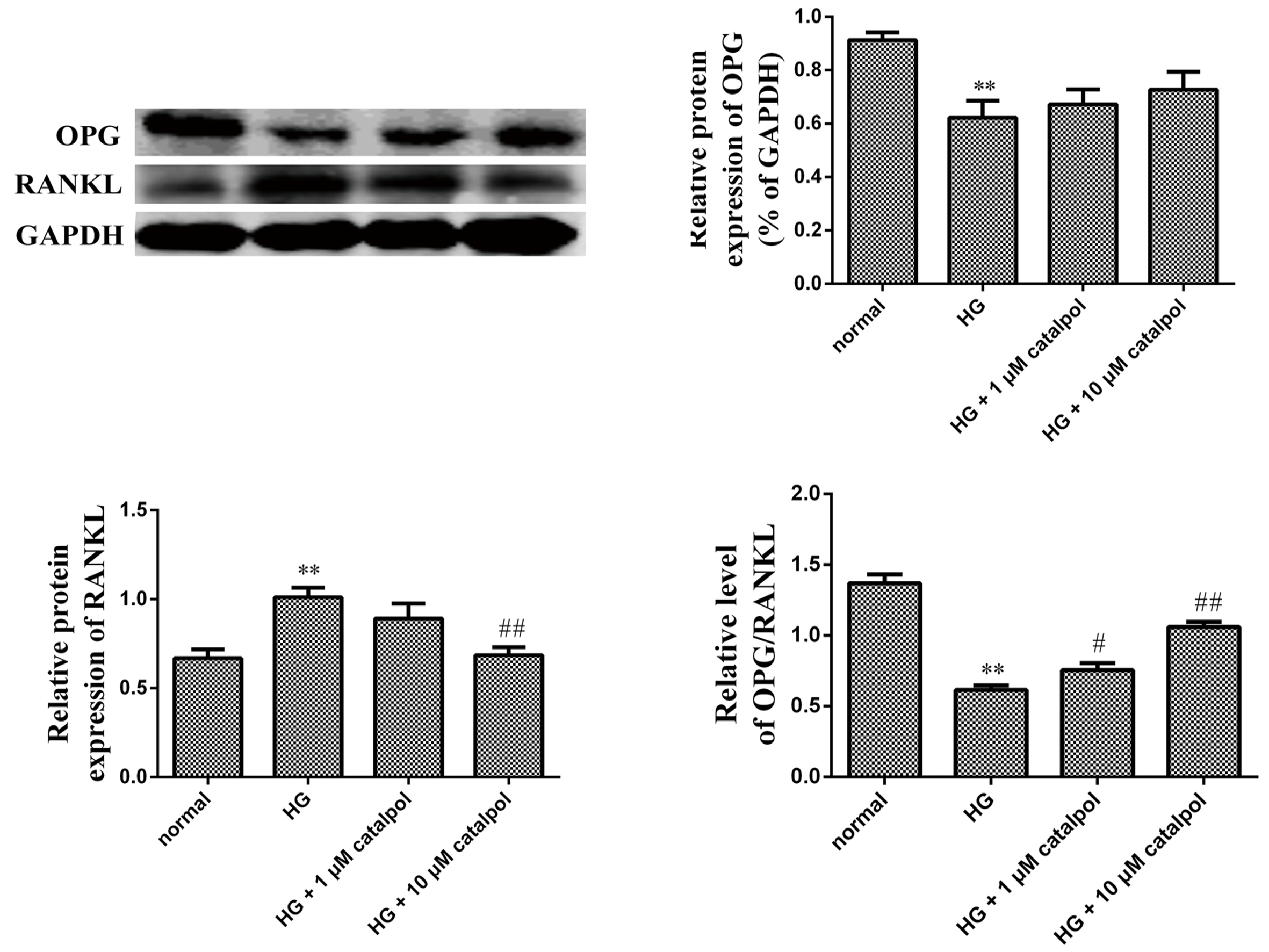

Supplement: Supplementary file 1 [file datasheet1.zip › Supplementary Material Presentation/Figure 6.tif]

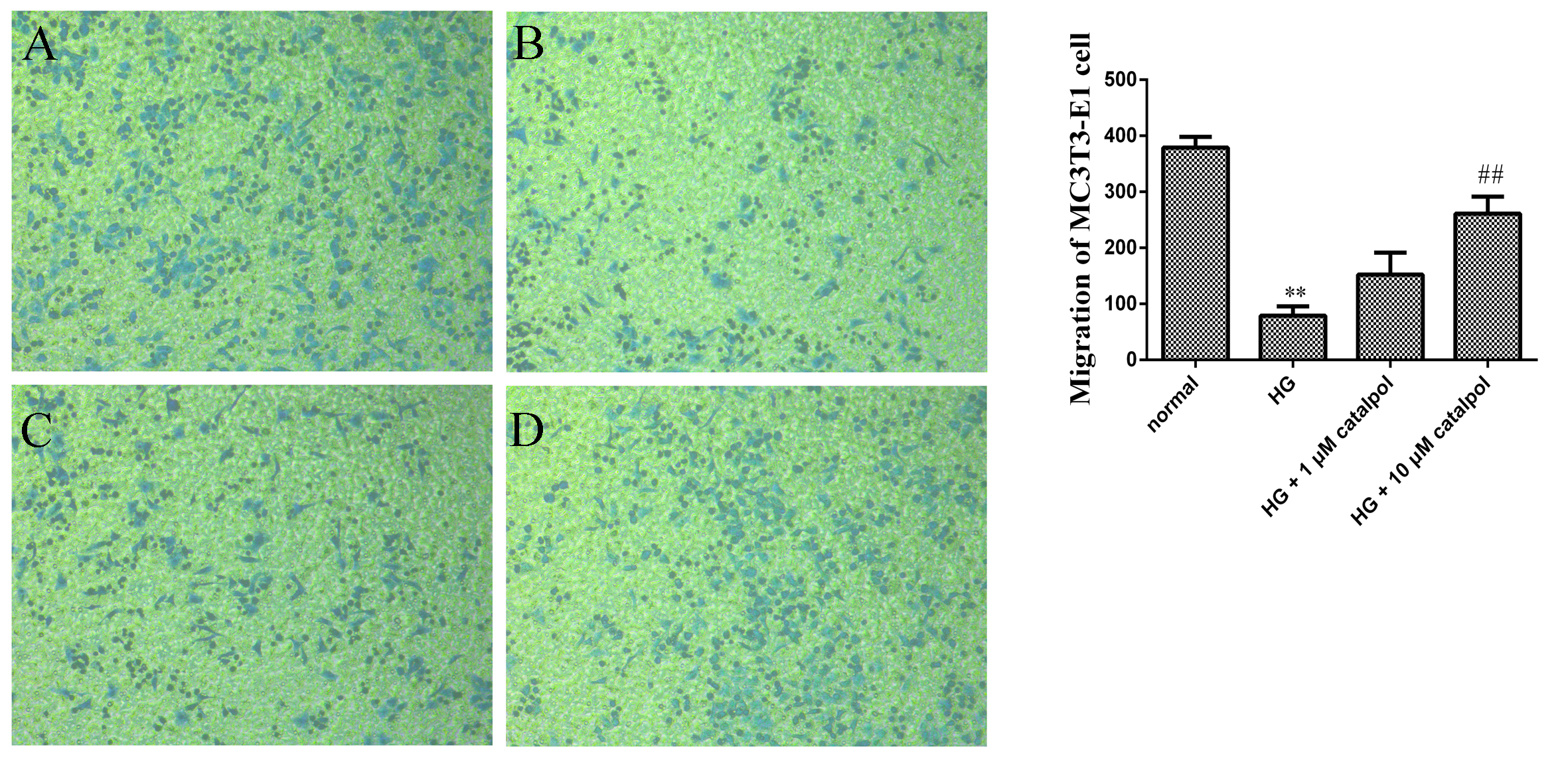

Supplement: Supplementary file 1 [file datasheet1.zip › Supplementary Material Presentation/Figure 7.tif]

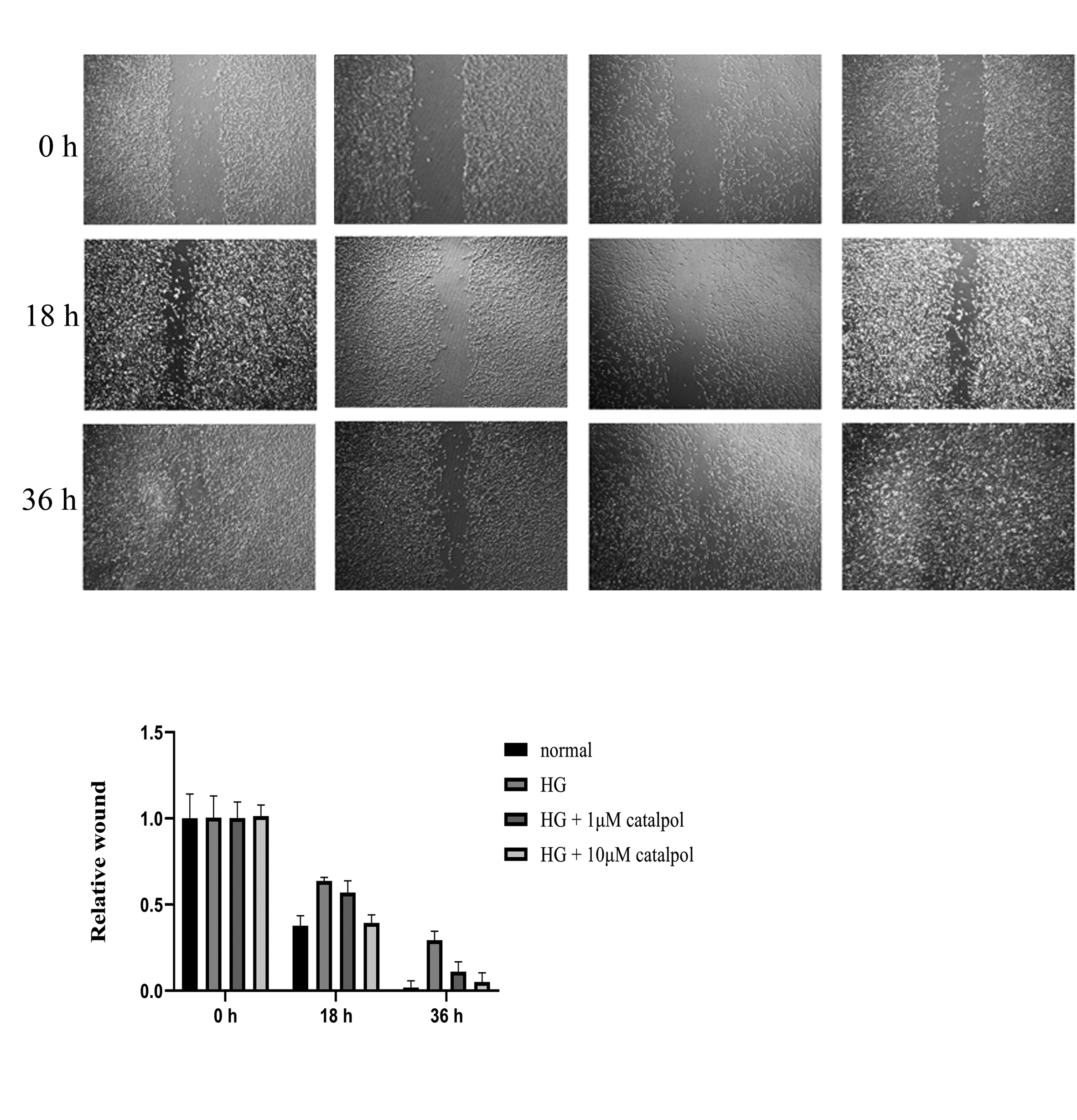

Supplement: Supplementary file 1 [file datasheet1.zip › Supplementary Material Presentation/Figure 8.tif]

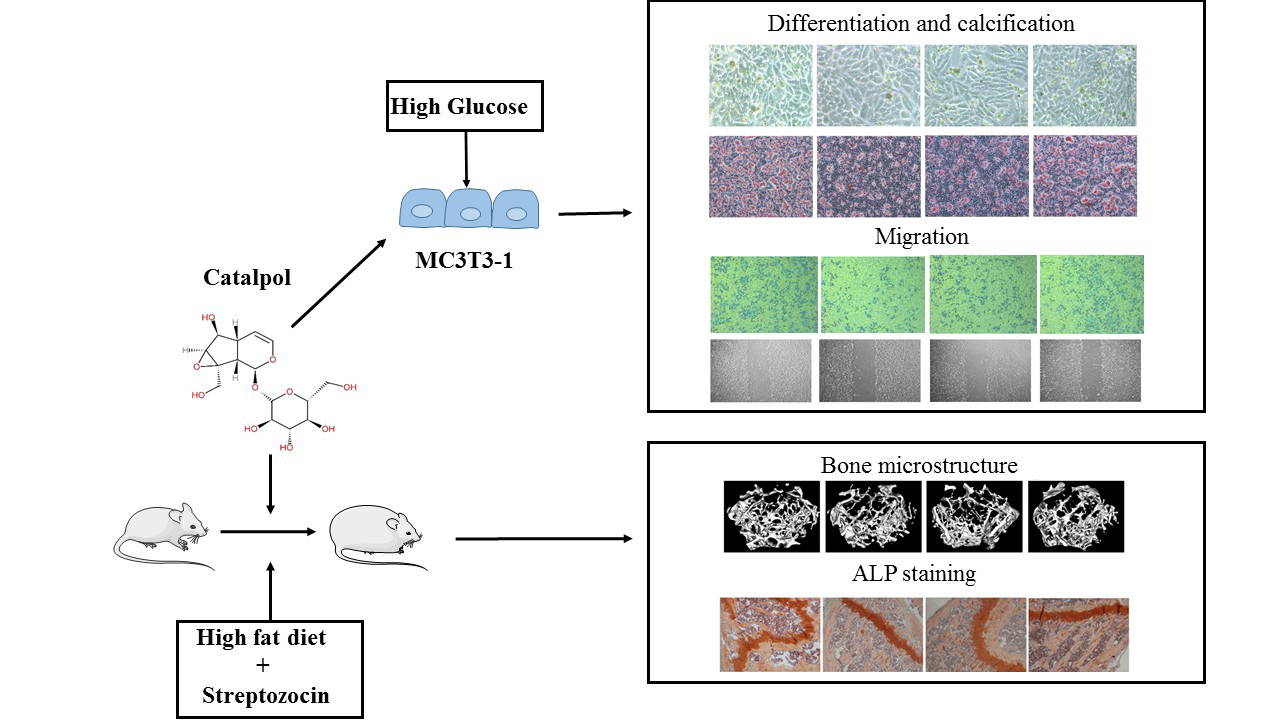

Supplement: Supplementary file 1 [file datasheet1.zip › Supplementary Material Presentation/graphical abstract.tif]

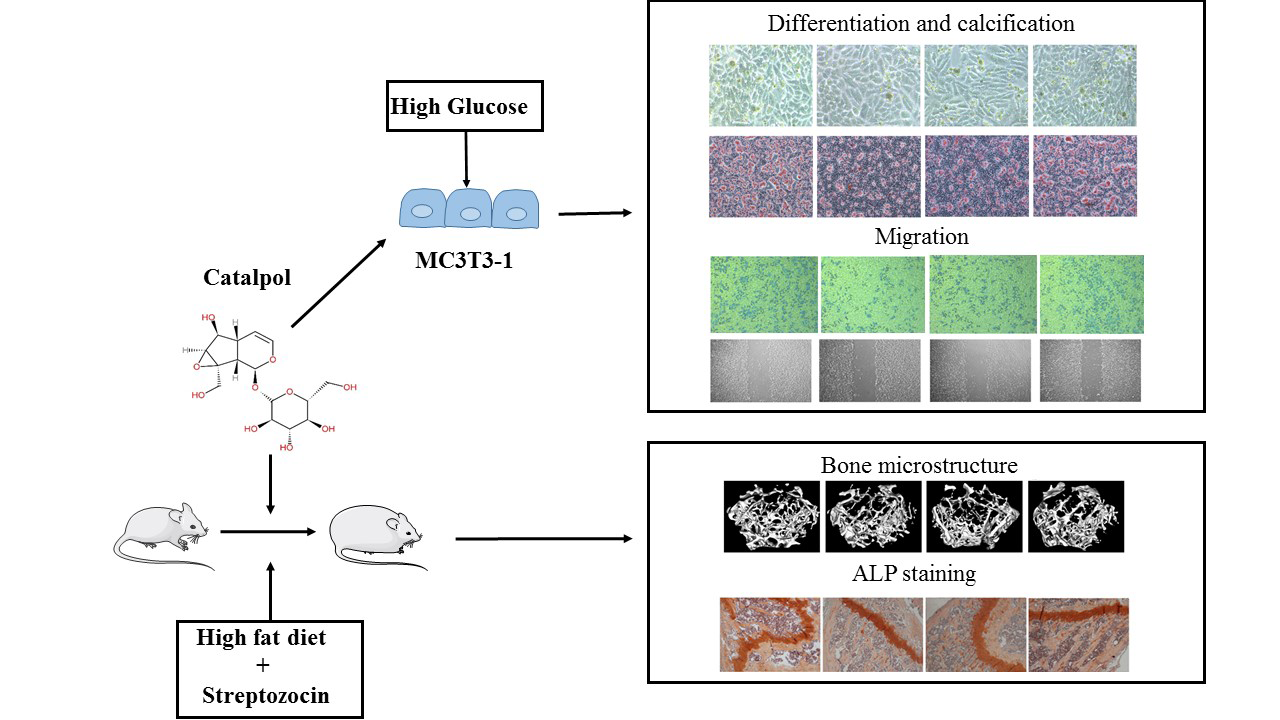

Supplement: Supplementary file 2 [file image1.tif]
